# Challenges in caring for people with dementia during COVID-19: Findings from long-term care facilities across Japan

**DOI:** 10.1093/geroni/igab046.2667

**Published:** 2021-12-17

**Authors:** Tomoko Ikeuchi, Mizue Suzuki, Kazunori Kikuchi, Akiko Kan, Chiho Shimada

**Affiliations:** 1 Tokyo Metropolitan Institute of Gerontology, Tokyo, Tokyo, Japan; 2 Hamamatsu Universuty school of medicne, Hamamatsu, Shizuoka, Japan

## Abstract

Long-term care facilities for older adults have been profoundly affected by the coronavirus disease 2019 (COVID-19) pandemic in Japan. This study investigated the challenges that care staff members faced by the height of the first wave (i.e., April 2020) and the height of the second wave (i.e., October 2020) of the pandemic at long-term care facilities in Japan. We mailed questionnaires in October 2020 to 5,895 care facilities throughout Japan. A total of 22.7% of the questionnaires were returned. Of those, 87.4% had at least one resident with dementia. Based on the results, 65.2% reported having restricted all visitors during the first wave. Although 42.8% reported continuing to restrict all visitors during the second wave, more than 54% allowed visitors while limiting the number of visitors or the time of each visit. Nearly 76% reported that restrictions on visitations may have exacerbated the behavioral and psychological symptoms of dementia (BPSD) among residents. In place of visitations, over 50% used video calls or phone calls to communicate with the family members, and 45.7% reported that the virtual visits were efficacious in alleviating the BPSD. However, more than 70% reported not having adequate Internet services and computer equipment at their facilities, and nearly 90% reported insufficient staffing. Our findings suggest that the pandemic may have pressed for a change of direction for dementia care with the introduction of virtual visitations and other initiatives. Nevertheless, facilities may face difficulties with implementing such changes due to inadequacies in the availability of resources.

